# Hypo-Expression of Tuberin Promotes Adenomyosis *via* the mTOR1-Autophagy Axis

**DOI:** 10.3389/fcell.2021.710407

**Published:** 2021-07-29

**Authors:** Ni-Hao Gu, Guo-Jing Li, Bing-Xin Yang, Min You, Yu Lin, Feng Sun, Hong Xu

**Affiliations:** ^1^International Peace Maternity and Child Health Hospital, School of Medicine, Shanghai Jiao Tong University, Shanghai, China; ^2^Shanghai Key Laboratory of Embryo Original Diseases, Shanghai, China; ^3^Shanghai Municipal Key Clinical Specialty, Shanghai, China

**Keywords:** adenomyosis, TSC2, autophagy, mTOR1, migration, EMT, endometrial cell

## Abstract

Adenomyosis (AM) is a disease in which endometrial tissue invades the myometrium and has a 10–60% prevalence in reproductive-aged women. TSC2 regulates autophagy *via* mTOR1 signalling in colorectal cancer and endometrial carcinoma. Dysregulation of autophagy is implicated in adenomyosis pathogenesis. However, whether TSC2 participates in adenomyosis *via* autophagy remains obscure. Here, we found that the expression of TSC2 in adenomyosis was significantly decreased than that in normal endometrium during the secretory phase. Moreover, TSC2 and autophagy marker expression was significantly lower in ectopic lesions than in eutopic samples. TSC2 downregulation inhibited autophagy through mTOR1 signalling pathway activation in endometrial cells, leading to excessive proliferation, migration, and EMT; TSC2 overexpression induced the opposite effects. Rapamycin treatment suppressed cell proliferation, migration and EMT in the absence of TSC2. In parallel, an autophagy-specific inhibitor (SAR-405) restored migration and EMT under rapamycin treatment in TSC2-knockdown Ishikawa cells. Finally, SAR-405 treatment promoted EMT and migration of overexpressing cells. Collectively, our results suggest that TSC2 controls endometrial epithelial cell migration and EMT by regulating mTOR1-autophagy axis activation and that hypo-expression of TSC2 in the endometrium might promote adenomyosis.

## Introduction

Adenomyosis (AM) is a benign uterine disorder characterised by the presence of ectopic endometrial tissue in the myometrium with hyperplasia of adjacent smooth muscle (Chapron et al., 2020). It is one of the leading disorders causing pelvic pain, abnormal uterine bleeding and subfertility and thus significantly impacts the quality of life in women of reproductive age (Leyendecker et al., 2002; Vercellini et al., 2014; Yang et al., 2021). Previous studies have demonstrated that gonadal hormone dependence/resistance, abnormal cell migration, and epithelial-mesenchymal transition (EMT) are involved in the pathogenesis of AM, but the precise mechanism underpinning AM remains unknown (Chen et al., 2010; Oh et al., 2013; Vannuccini et al., 2017; Zhou et al., 2018; Hu et al., 2020).

Autophagy is a critical cellular process that enables scavenging and recycling of non-essential proteins and organelles to maintain cellular homoeostasis (Mizushima et al., 2008). Accumulated evidence shows that autophagy participates in the regulation of the endometrial cell cycle and that its dysregulation promotes carcinogenesis in many tissues through augmentation of cellular proliferation, migration and EMT processes (Choi et al., 2014; Gugnoni et al., 2017; Liu et al., 2020; Zhao et al., 2020). In addition, it has been suggested that aberrant autophagy occurs in endometrial cells in patients with AM (Ren et al., 2010). Prior studies have shown that the expression of LC3B-II, which is an indicator of autophagic activity, is elevated in the secretory phase in normal endometrial tissues but not in AM endometrial tissues during either the proliferative or secretory phase (Li et al., 2018). Thus far, most of studies have supported the opinion that autophagy dysfunction exists in ectopic endometrial cells due to a hyper-estrogenic state (Mei et al., 2015; Pei et al., 2019). Although there is a lack of direct evidence that abnormal autophagy is involved in progesterone (P_4_) resistance in ectopic endometrial cells, mammalian target of rapamycin (mTOR), a putative upstream signalling component of autophagy, is overactivated in progesterone-resistant endometrial cancer cells (Liu et al., 2017). However, the precise molecular mechanisms of AM are incompletely understood.

Tuberin (TSC2), a tumour suppressor, regulates the activity of mTOR that regulates protein synthesis, cell growth, and autophagy (Kwiatkowski and Manning, 2014; Li et al., 2014; Kim and Guan, 2015; Henske et al., 2016). Some pioneering studies have reported that dysregulation of TSC2 is associated with female infertility and reproductive tract cancer (Sales et al., 2004; Ju et al., 2007; Lu et al., 2008; Adhikari et al., 2009; Reddy et al., 2010). Therefore, we hypothesised that aberrant expression of TSC2 in endometrial cells hinders autophagy induction through regulation of mTOR activation, leading to the occurrence of AM.

In the present study, we first described the dysrhythmic expression of TSC2 in the endometria of individuals with AM during the menstrual cycle. We then detected hypo-expression of TSC2 in ectopic lesions compared with eutopic endometrium that was positively related to the expression pattern of LC3A. When TSC2 was knocked down in endometrial cells, over-activation of mTOR and/or impairment of autophagy induction induced excessive proliferation, migration, and EMT. Eventually, cellular proliferation and migration were restored to normal levels after treatment with antagonists of mTOR and autophagy in pharmacological experiments. Collectively, our findings indicate that TSC2 inhibits the abnormal migration and EMT of endometrial cells by regulating the mTOR-autophagy axis.

## Results

### Demographics

Table 1 summarises the demographic characteristics of the study subjects; there were no significant differences between the two groups except for the clinical symptoms of AM.

**TABLE 1:** Characteristics of recruited patients with AM and controls.

Characteristic	Control group (*n* = 20)	Adenomyosis group (*n* = 20)	*P* value
**Age** (years; median [IQR])	49 (5)	47.5 (3)	0.784
**Menstrual phase**			
Proliferative	8 (40.0%)	8 (40.0%)	1.000
Secretory	12 (60.0%)	12 (60.0%)	
**Gravidity**			0.556
0	0 (0.0%)	2 (10.0%)	
1	7 (35.0%)	5 (25.0%)	
2	7 (35.0%)	5 (25.0%)	
≥3	6 (30.0%)	8 (40.0%)	
**Parity**			0.02
0	0 (0.0%)	6 (30.0%)	
1	18 (90.0%)	13 (65.0%)	
2	1 (5.0%)	1 (5.0%)	
≥3	1 (5.0%)	0 (0.0%)	
**Severity of dysmenorrhoea**			<0.001
None	14 (70.0%)	0 (0.0%)	
Mild	5 (25.0%)	3 (15.0%)	
Moderate	1 (5.0%)	6 (30.0%)	
Severe	0 (0.0%)	11 (55.0%)	
**VAS score (median [IQR])**	0 (2)	7.5 (4)	<0.001
**Uterus size, in cm** ^3^	47.6 (19.6)	229.7 (151.9)	<0.001

Uterus size calculated as (π * D1 * D2 * D3)/6, where D1 = the distance from fundus to the internal os of the cervix, D2 = transverse diameter at the level of the cornua, and D3 = anteroposterior diameter at the level of cornua.

*Data are presented as the median (IQR) or number (percentage).*

*VAS means Visual Analogue Scale; IQR means interquartile range.*

### TSC2 Is Rhythmically Expressed in the Eutopic Endometrium During the Menstrual Cycle, but Not in Patients With AM

**FIGURE 1:**
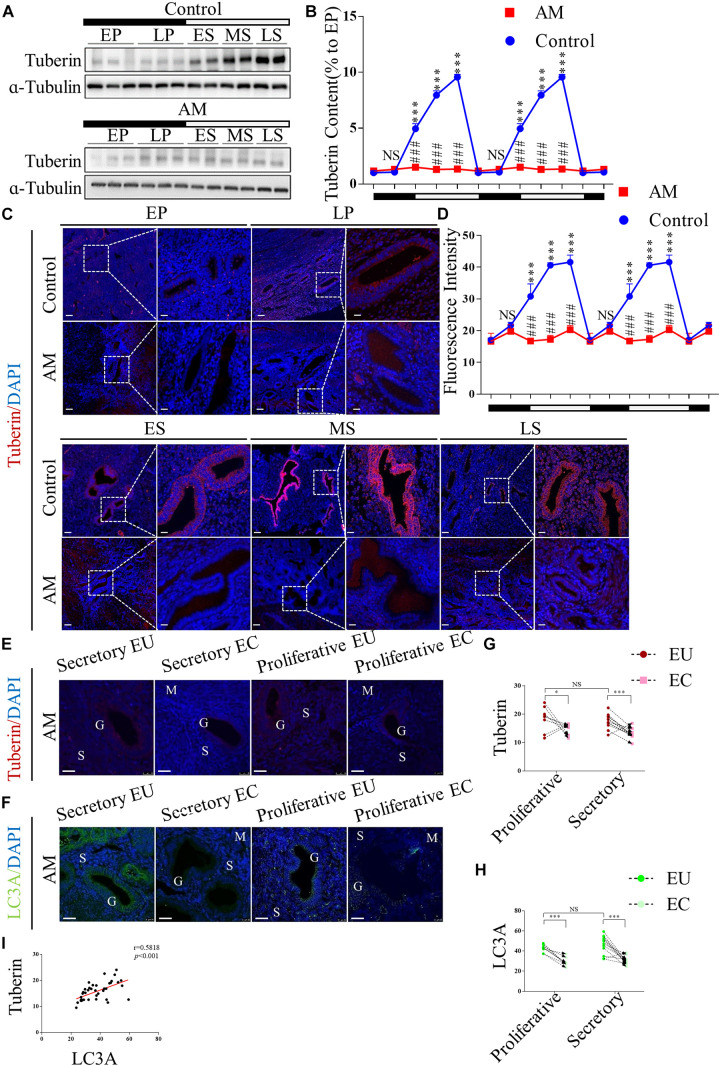
Tuberin (TSC2) is dysrhythmic hypo-expressed in patients with adenomyosis (AM) during the menstrual cycle, and correlated with the expression of LC3 in both the eutopic and ectopic endometria. **(A)** Representative immunoblots of TSC2 in the endometrial tissues of healthy women and patients with AM. EP, early proliferative phase; LP, late proliferative phase; ES, early secretory phase; MS, mid-secretory phase; LS, late secretory phase. The black part of the timeline represents the proliferative phase (with the early and late proliferative phases sequentially indicated); the white part represents the secretory phase (with the early, mid-, and late secretory phases sequentially indicated). **(B)** Relative protein expression of TSC2 in panel **(A)**. The data are double plotted for better visualisation. The black part of the timeline represents the proliferative phase (with the early and late proliferative phases sequentially indicated); the white part represents the secretory phase (with the early, mid-, and late secretory phases sequentially indicated). NS *p* > 0.05 and ^∗∗∗^*p* < 0.001 compared with the early proliferative phase. NS *p* > 0.05 and ###*p* < 0.001 compared with the control group. **(C)** Representative immunofluorescence images of TSC2 in the endometrial tissues of healthy women and patients with AM. **(D)** Fluorescence intensity of the TSC2 expression in panel **(C)**. Scale bars, 25 μm. NS *p* > 0.05 and ^∗∗∗^*p* < 0.001 compared with the early proliferative phase. NS *p* > 0.05 and ###*p* < 0.001 compared with the control group. **(E–H)** The expression levels of TSC2 and LC3A in eutopic endometria (EU) are higher than those in ectopic tissues (EC) in both the secretory and proliferative phases. NS *p* > 0.05, ^∗^*p* < 0.05, and ^∗∗∗^*p* < 0.001 compared with the EU group. G, glandular epithelium; S, stroma; M, smooth muscle. Scale bar, 25 μm. **(I)** Correlation between TSC2 and LC3A expression (*p* < 0.001, *r* = 0.58). The data are presented as the mean ± SEM.

To investigate whether TSC2 is expressed and plays a role in human endometria with or without AM, we collected human endometrial biopsies from 40 subjects and analysed the expression of TSC2 using western blotting and immunofluorescence (IF) staining. In the normal tissues (*n* = 20), as shown in Figures 1A,B, the expression of TSC2 increased after the late proliferative phase and was significantly higher in the early, mid-, and late secretory phases than in the early proliferative phase. Subsequently, we evaluated the expression of TSC2 in AM eutopic endometrial tissues. As shown in Figures 1A,B, TSC2 was hypo-expressed in all phases during the menstrual cycle.

Consistently, IF staining showed that TSC2 was widely expressed in the epithelial cells of normal endometrial tissue and localised in the cytoplasm in all phases of the menstrual cycle (Figure 1C). The fluorescence intensity of TSC2 in the normal tissues was significantly increased in the secretory phase (Figure 1D). However, TSC2 fluorescence intensity was significantly lower in AM endometrial tissues than in normal endometrial tissues during the secretory phase (Figure 1D). To verify the above hypothesis, we also detected the expression of TSC2 in AM tissues, including 20 pairs of ectopic and eutopic specimens, using IF staining (Figure 1E). The expression of TSC2 in lesions was significantly downregulated compared with that in their eutopic counterparts (Figure 1G).

These results indicate that rhythmic expression of TSC2 may participate in the remodelling of the human endometrium during the menstrual cycle and that hypo-expression of TSC2 may contribute to the occurrence of AM.

### The Expression Level of MAP1LC3A Is Positively Correlated With the Expression of TSC2 in Human AM Samples

MAP1LC3A (LC3A) is an authentic autophagy marker in mammalian cells that is homologous to Atg8 in yeast. Thus, we examined the expression of LC3A in our AM tissues, and the results showed that LC3A expression was lower in lesional tissues than in eutopic tissues (Figures 1F,H). Linear regression analysis showed that low TSC2 expression was positively correlated with LC3A expression (Figure 1I).

### Knockdown of TSC2 Suppresses Autophagy Induction and Enhances Proliferation in Ishikawa Cells

**FIGURE 2:**
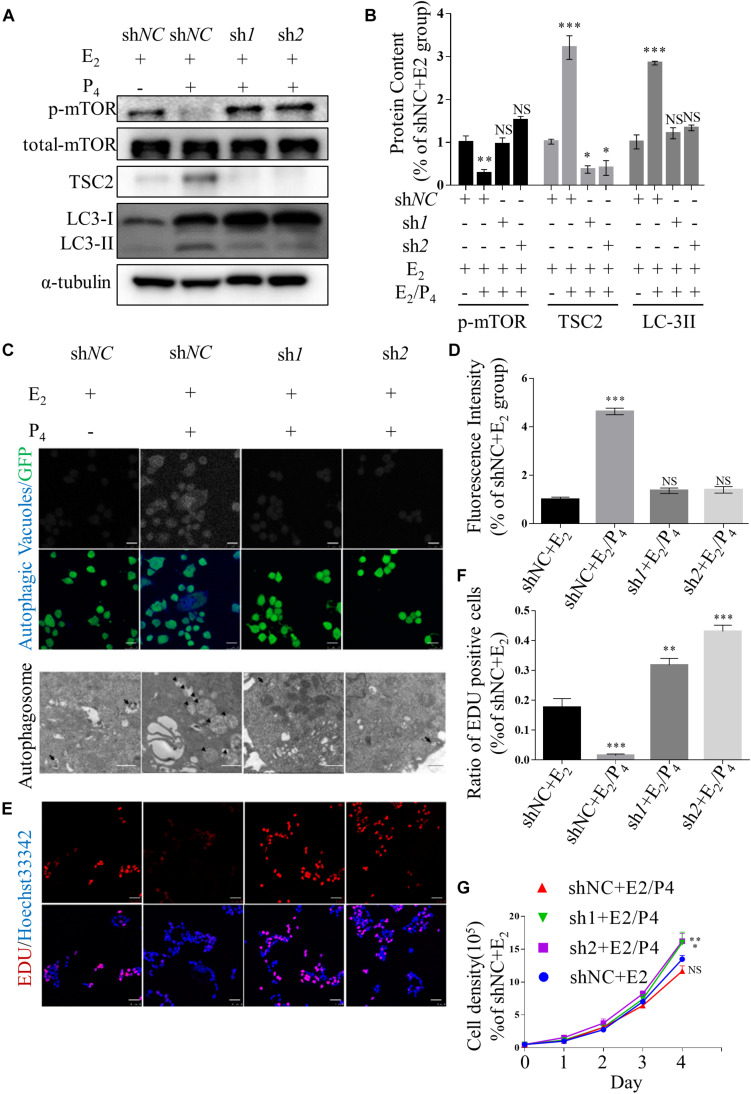
Knockdown of TSC2 suppresses autophagy and increases cell proliferation. **(A,B)** Representative immunoblots and quantification of LC3, phosphorylated mTOR1 and TSC2 in Ishikawa cells, which had been TSC2 knock-out (sh*1* and sh*2*, sh*NC* as control), treated with E_2_ and/or P_4_ or then subjected to withdrawal. **(C)** Typical images of autophagic vacuoles detected using immunofluorescence staining and transmission electron microscopy (TEM) (the arrows indicate representative autophagosomes). sh*1* and sh*2* as TSC2-knockdown Ishikawa cells and sh*NC* as control. Immunofluorescence scale bars, 25 μm; TEM scale bars, 1 μm. **(D)** Fluorescence intensity of the autophagic vacuoles in panel **(C)**. **(E)** An EdU incorporation assay showed that cell proliferation was increased after depletion of TSC2. Scale bars, 50 μm. **(F)** Statistical results showing the percentages of EdU-positive cells in the assay. **(G)** Cell growth curves of Ishikawa cells under various conditions. The data are presented as the mean ± SEM. NS *p* > 0.05, ^∗^*p* < 0.05, ^∗∗^*p* < 0.01, and ^∗∗∗^*p* < 0.001 compared with the shNC + E_2_ group.

To investigate the role of TSC2 in endometrial cells, we used lentiviruses harbouring TSC2 shRNA to knockdown TSC2 in Ishikawa cells. First, western blotting validated that the expression of TSC2 was effectively decreased by TSC2-targeted shRNA (Figures 2A,B). Immunofluorescence showed that autophagy induction in Ishikawa cells was significantly decreased after treatment with P_4_ in the absence of TSC2 (Figures 2C,D). Moreover, transmission electron microscopy showed that the numbers of autophagosomes were decreased in TSC2 shRNA- infected cells (Figure 2C). As expected, western blot analysis showed that downregulation of TSC2 in Ishikawa cells inhibited the expression of LC-3II/LC-3I (Figures 2A,B). Furthermore, endometrial cell growth curves and EdU incorporation assay results were analysed after knockdown of TSC2. The assays revealed that cell proliferation was increased after TSC2-targeted shRNA treatment (Figures 2E–G). These findings suggest that TSC2 might be induced by P_4_ and regulate autophagy induction and proliferation *in vitro*.

### Knockdown of TSC2 Promotes Cellular EMT and Migration

**FIGURE 3:**
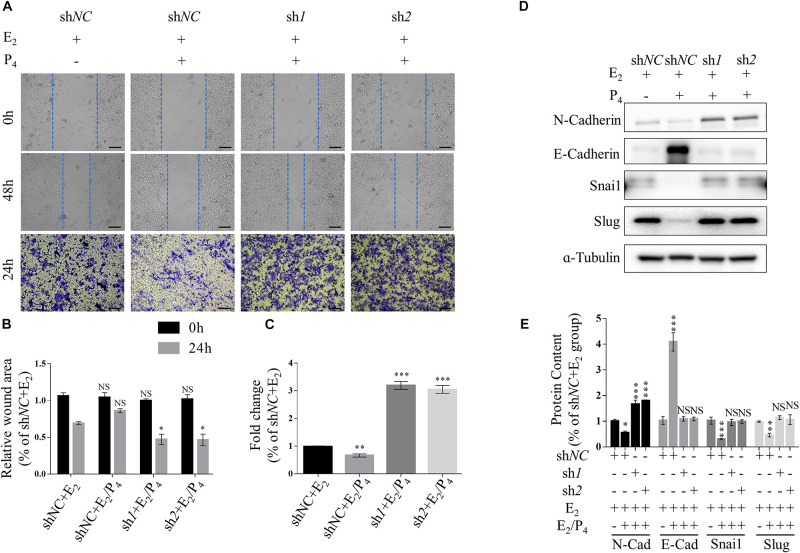
Knockdown of TSC2 promotes epithelial-mesenchymal transition (EMT) and migration. **(A–C)** Representative images and statistics of cells in the scratch assay and Transwell assay. Scale bars in **(A,B)**, 100 μm. **(D,E)** Representative immunoblot and quantification of the expression of N-Cadherin, E-Cadherin, Snail and Slug in Ishikawa cells following treatment with E_2_, treatment with E_2_ and P_4_ or withdrawal of these hormones in culture medium. The data are presented as the mean ± SEM. NS *p* > 0.05, ^∗^*p* < 0.05, ^∗∗^*p* < 0.01, and ^∗∗∗^*p* < 0.001 compared with the shNC + E_2_ group.

Transwell migration assay and scratch wound healing assay were performed to determine the effects of TSC2 on cellular migration. These assays showed that knockdown of TSC2 enhanced migration of Ishikawa cells (Figures 3A–C). Following 24 h of culture, TSC2 shRNA-infected Ishikawa cells had travelled significantly farther than control cells. Finally, western blot analysis showed increased expression of N-Cadherin, Snail and Slug and decreased expression of E-Cadherin in the shRNA groups (Figures 3D,E). These findings demonstrate that TSC2 regulates endometrial cell migration, maintains E-Cadherin expression and reduces N-Cadherin, Snail and Slug expression, restricting EMT of endometrial cells in normal tissues.

### Overexpression of TSC2 Promotes Autophagy Induction and Impairs Proliferation in Endometrial Cells

To further elucidate the role of TSC2, we enhanced the expression of TSC2 using a TSC2 overexpression plasmid in Ishikawa cells. Western blot analysis showed that the TSC2 plasmid successfully induced overexpression (Supplementary Figures 2A,B). Commensurately, the expression of phosphorylated mTOR was significantly lower and the LC3-II/LC3-I ratio was higher in TSC2-overexpressing cells than in mock cells cultured under oestradiol (E_2_) treatment.

As autophagy induction in endometrial cells is an important cyclic activity during the menstrual cycle, we investigated the effect of TSC2 overexpression on autophagy induction in endometrial cells. Western blot, IF and TEM assays showed increased levels of autophagy induction in TSC2-overexpressing Ishikawa cells (Supplementary Figures 3A,B). Moreover, the results from EdU incorporation and cell density assays showed that cell proliferation was inhibited after TSC2 overexpression (Supplementary Figures 3A,C,D). In addition, significantly fewer cells passed through the chamber in the TSC2 overexpression group than in the mock group (Supplementary Figure 4A), consistent with the findings of the scratch wound healing assay (Supplementary Figures 4B,C). Finally, N-Cadherin, Snail and Slug expression was decreased in TSC2-overexpressing cells compared with mock cells, while E-Cadherin expression was increased, indicating that EMT was inhibited in the TSC2-overexpressing cells (Supplementary Figures 4D,E).

### EMT and Migration Are Inhibited When Autophagy Induction Is Increased Using Rapamycin *in vitro*

To determine whether the proliferation, EMT and migration of TSC2-overexpressing and TSC2-knockdown cells are mediated by autophagy, we evaluated the effects of rapamycin and SAR-405, a specific mTOR1 inhibitor and a specific vacuolar protein sorting 34 (VPS34) inhibitor, respectively, in the presence of E_2_ or E_2_/P_4_.

**FIGURE 4:**
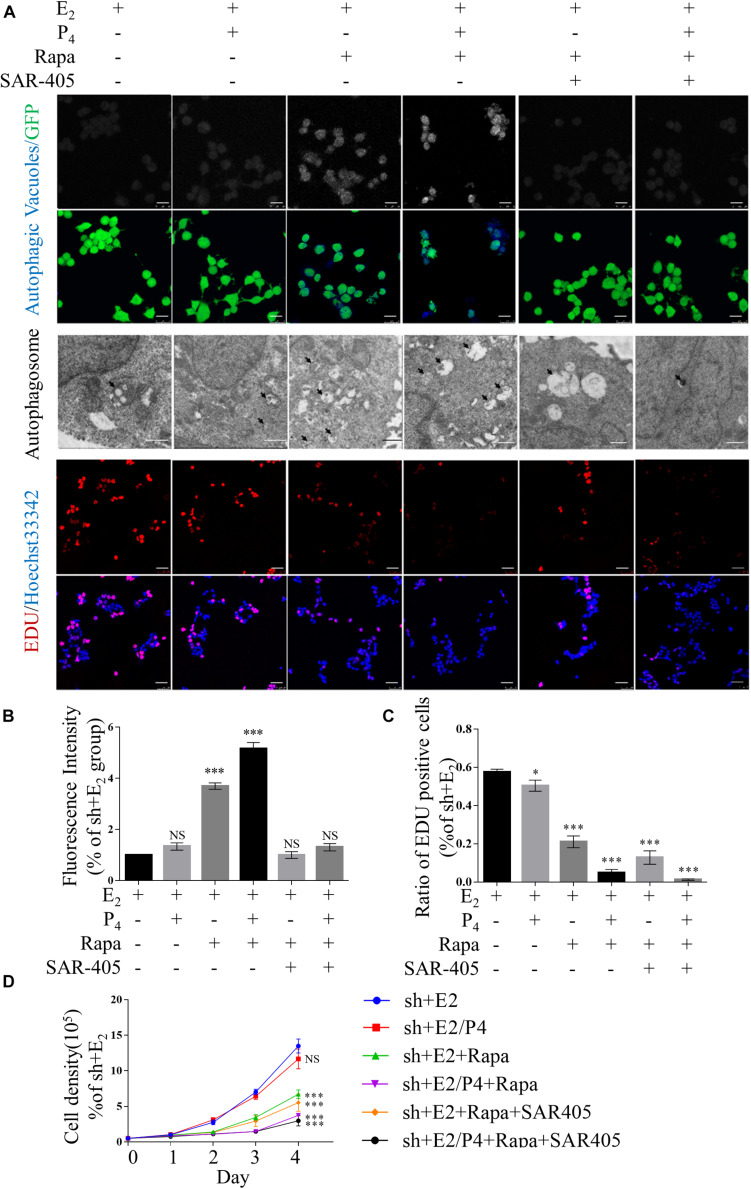
Defects in autophagy and hyper-proliferation were reversed by mTOR inhibition *in vitro*. **(A)** Typical images of autophagic vacuoles detected using immunofluorescence staining and transmission electron microscopy (TEM) (the arrows indicate autophagosomes) and EdU-positive cells. Immunofluorescence scale bars, 25 μm; TEM scale bars, 1 μm; EdU scale bars, 50 μm. **(B)** Fluorescence intensity of autophagic vacuoles in panel **(A)**. **(C)** Statistical results showing the percentage of EdU-positive cells in the EdU incorporation assay in panel **(A)**. **(D)** Cell growth curves of Ishikawa cells under various conditions. The data are presented as the mean ± SEM. NS *p* > 0.05, ^∗^*p* < 0.05, and ^∗∗∗^*p* < 0.001 compared with the shNC + E_2_ group.

Supplementary Figures 5A,B show that the expression of phosphorylated mTOR was significantly lower in rapamycin-treated cells compared with mock cells in the presence of P_4_. In addition, the expression levels of LC3-II/LC-3I were increased in TSC2-knockdown cells. Moreover, LC3-II/LC-3I expression was suppressed significantly by treatment with both rapamycin and SAR-405. As shown in Figures 4A,B, rapamycin treatment significantly increased the number of autophagic cells in the presence of P_4_. In contrast, the proportion of autophagic rapamycin-treated cells was significantly decreased after addition of SAR-405. As in TSC2-overexpressing cells, decreased expression of LC3-II/LC3-I were detected after SAR-405 treatment (Supplementary Figures 2A,B).

In addition, after TSC2-knockdown Ishikawa cells were treated with rapamycin, cell proliferation was suppressed (Figures 4A,C,D). However, cell proliferation was not changed by the addition of SAR-405 (Figures 4A,C,D). Under the same culture conditions, SAR-405 treatment did not rescue the cell proliferation ability of TSC2-overexpressing cells (Supplementary Figures 3A,C,D). These results indicate that cell proliferation is directly regulated by mTOR but not autophagy induction.

**FIGURE 5:**
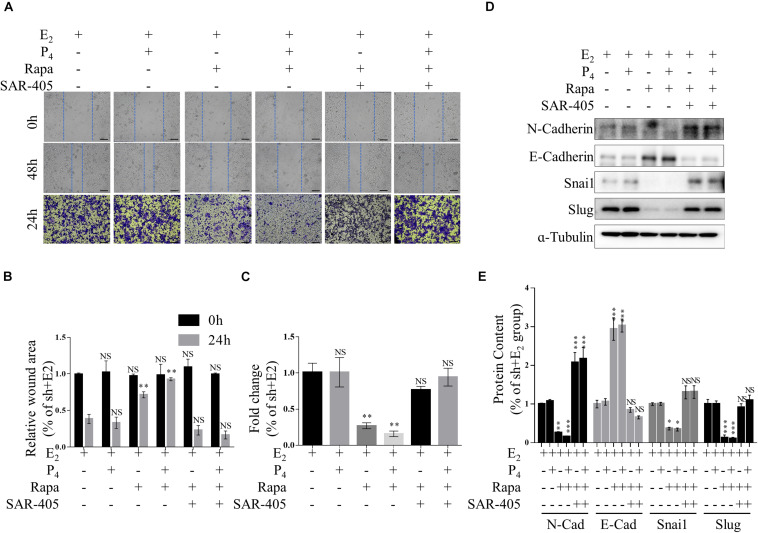
mTOR inhibition ameliorates increased EMT and migration by increasing autophagy *in vitro*. **(A–C)** Representative images **(A,B)** and statistics **(C)** of cells in the scratch assay and Transwell assay. Scale bars in **(A,B)**, 100 μm. **(D,E)** Representative immunoblot and quantification of N-Cadherin, E-Cadherin, Snail and Slug expression in TSC2-knockdown Ishikawa cells treated with rapamycin or rapamycin + SAR-405. The data are presented as the mean ± SEM. NS *p* > 0.05, ^∗^*p* < 0.05, ^∗∗^*p* < 0.01, and ^∗∗∗^*p* < 0.001 compared with the shNC + E_2_ group.

Furthermore, we detected the migration of rapamycin-treated TSC2 shRNA- infected cells using both Transwell migration assay and scratch wound healing assay. When the cells were treated with rapamycin, the gap in the TSC2-knockdown group was significantly larger than that in the control group (Figures 5A–C). Intriguingly, the TSC2 shRNA- infected cells that were treated with both rapamycin and SAR-405 regained their hyper-migration ability (Figures 5A–C). Moreover, rapamycin-treated cells exhibited reduced N-cadherin, Snail, and Slug protein expression and increased E-cadherin protein expression (Figure 5D). Importantly, SAR-405 was able to completely restore the hyper-EMT property by repressing autophagy (Figures 5D,E). Moreover, SAR-405 enhanced the EMT status and migration of TSC2-overexpressing endometrial cells in either proliferative or secretory phases (Supplementary Figure 4).

### Effects of Oestrogen(E_2_) and Progesterone(P_4_) on Autophagy and mTOR Activity in Human Endometrial Epithelial Cells (HEECs) and Ishikawa Cells *in vitro*

**FIGURE 6:**
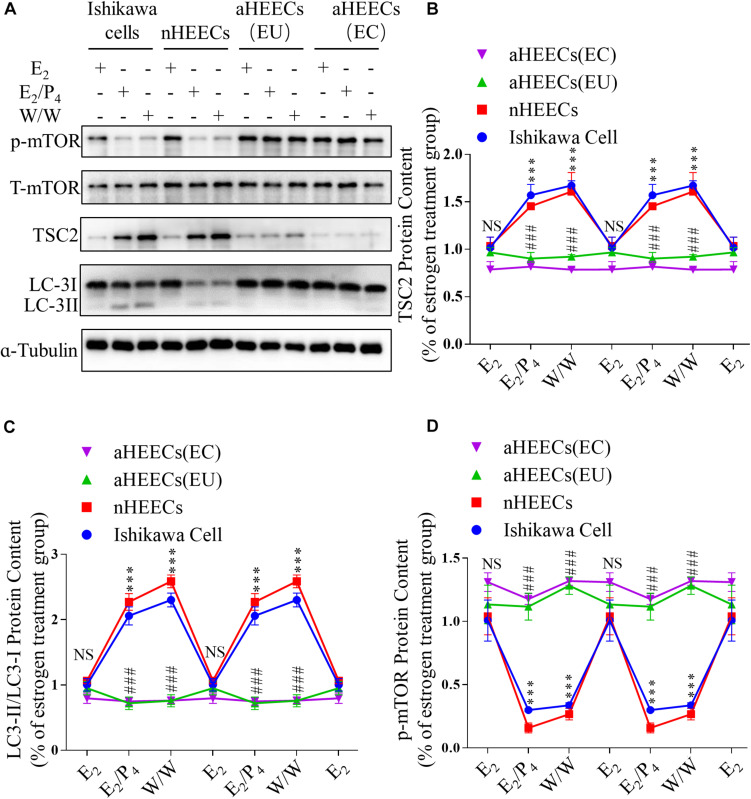
Primary endometrial cells from patients with AM lack responsiveness to P4 and E_2_ in the TSC2-mTOR-autophagy axis. **(A)** Representative immunoblots of LC3, phosphorylated mTOR1 and TSC2 in Ishikawa cells, nHEECs and aHEECs (from ectopic endometrium or eutopic endometrium, respectively). **(B–D)** Statistical results showing the loss of rhythmic expression of TSC2 **(B),** LC-3 **(C)**, and phosphorylated mTOR **(D)** in primary endometrial cells from patients with AM. The data are double-plotted for better visualisation. The data are presented as the mean ± SEM. ^∗^*p* < 0.05, ^∗∗^*p* < 0.01, and ^∗∗∗^*p* < 0.001 compared with the E_2_ group. NS *p* > 0.05, #*p* < 0.05, and ##*p* < 0.01 compared with the Ishikawa cell group.

To further determine whether ovarian steroids regulate TSC2 expression and subsequent mTOR activity and autophagy induction, we isolated glandular epithelial cells from the control subjects and AM patients and examined the effects of E_2_ and P_4_ on TSC2, LC3-II/LC3-I, and phosphorylated mTOR expression in cultured normal HEECs (nHEECs) and AM HEECs (aHEECs). The results presented in Figures 6A,B show that the expression levels of TSC2 were significantly higher in nHEECs cultured with P_4_ or subjected to withdrawal than in nHEECs cultured with E_2_ alone. Furthermore, the expression of LC3-II/LC3-I was significantly higher, while that of phosphorylated mTOR was lower, in nHEECs cultured with E_2_ and P_4_ than in nHEECs cultured with E_2_ alone.

In aHEECs, however, E_2_ and P_4_ failed to change the expression levels of TSC2, phosphorylated mTOR and LC3-II/LC3-I (Figure 6A). Compared with nHEECs, aHEECs exhibited significantly lower TSC2 and LC3-II/LC3-I expression and higher phosphorylated mTOR expression (Figures 6A–D).

In addition, we also detected similar effects of ovarian steroids on autophagy and mTOR activity in Ishikawa cells (Figure 6A). The expression levels of TSC2 were significantly higher in cultured Ishikawa cells treated with P_4_ or undergoing withdrawal from both E_2_ and P_4_ than in Ishikawa cells cultured with E_2_ alone. The patterns of LC3-II/LC3-I and phosphorylated mTOR expression in Ishikawa cells treated with E_2_ and P_4_ were similar to those in nHEECs.

## Materials and Methods

### Ethical Approval

Ethical approval for the tissue collection was obtained from the ethics committee of the International Peace Maternity and Child Health Hospital affiliated with the School of Medicine, Shanghai Jiao Tong University. Written informed consent was obtained from all participants.

### Tissue Collection

Eutopic and ectopic endometrial tissues were collected from 20 patients (age range, 40–50 years) undergoing hysterectomy for progressive dysmenorrhoea only (*n* = 10), menorrhagia only (*n* = 2), or progressive dysmenorrhoea and menorrhagia (*n* = 8).

Healthy endometrial samples were obtained from 20 patients (age range, 38–53 years) with cervical carcinoma *in situ* undergoing laparoscopy.

The exclusion criteria for participants were endometrial abnormalities, pelvic endometriosis, fibroids, ovarian cysts, lesions, other obvious internal medical or surgical comorbidities and use of any steroid hormone therapy within the last 3 months. Each tissue specimen was separated into two parts: one part was frozen in liquid nitrogen for protein-related assays, and the other was fixed in 4% formalin for IF analysis.

According to the day of the menstrual cycle, the endometrial tissue samples were classified into five phases: the early proliferative phase (days 1–5), the late proliferative phase (days 6–14), the early secretory phase (days 15–18), the mid-secretory phase (days 19–23), and the late secretory phase (days 24–28).

### Cell Culture and Treatment

The Ishikawa cell line was a gift from the Shanghai Key Laboratory of Embryo Original Diseases. Primary HEECs were isolated as previously described (Guo et al., 2018). Briefly, primary cells were seeded at a density of 0.5 × 10^5^ cells/well in 24-well culture plates. The purity of the endometrial epithelial cells was validated by immunostaining with an E-cadherin antibody. All the cells were cultured in DMEM/F12 medium (11039021; Gibco, Logan, UT, United States) supplemented with 10% foetal bovine serum (FBS, 10099; Invitrogen, Carlsbad, CA, United States) and 1% penicillin/streptomycin (10378016; Gibco). The cells were incubated at 37°C in humidified air with 5% CO_2_ and harvested by brief incubation in 0.25% trypsin-EDTA (15400054; Gibco).

To mimic physiological hormonal changes, the cells were cultured with EBSS medium for 24 h following treatment with 10 nM oestradiol (E2758; Sigma-Aldrich, St. Louis, MO, United States) or 10 nM oestradiol (E2758; Sigma) + 1 mM progesterone (P0130; Sigma) for 24 h. For the with/withdrawal (W/W) group, the culture medium of the hormone-treated cells was replaced with hormone-free medium 6 h before analysis. Additionally, Ishikawa cells were cultured with either 1 mM rapamycin (V900930; Sigma) or 1 mM rapamycin + 5 μM SAR-405 (S7682; Selleck, Shanghai, China) to inhibit mTOR1 activity and autophagy, respectively. 6 h later, the medium was removed, and the cells were collected for further analysis.

### Viral Infection and Plasmid Transfection

Lentiviral transfection was performed as described previously (Li et al., 2020). To examine the function of TSC2 *in vitro*, Ishikawa cells (used as a model of epithelial cells) were infected with TSC2 shRNA-expressing lentiviruses provided by GenePharma (Shanghai, China) at a multiplicity of infection (MOI) of 10 for 72 h. The target sequences of the TSC2 shRNAs were as follows: 5′-GGATTACCCTTCCAACGAAGA-3′ and 5′-GGGACATTCTGCTGAACATCA-3′. The scramble shRNA sequence was 5′-GCACCTCTACAGGAACTTTGC-3′.

pcDNA3-TSC2 was a gift from Brendan Manning (plasmid #14129; Addgene) (Manning et al., 2002). Cells were transfected with plasmids using Lipofectamine 3000 transfection reagent (L3000015; Invitrogen) according to the manufacturer’s protocol. Briefly, cells were seeded at a density of 2 × 10^6^ cells/well in 6-well plates and incubated overnight. At 70–80% confluence, 2.5 μg of plasmid, 5 μL of P3000 and 7.5 μL of Lipofectamine 3000 were diluted with 250 μL of Opti-MEM Medium (31985088; Gibco) and incubated at room temperature for 15 min. The transfection complex was then added to the cells in 1.75 mL of complete medium per well. The medium was replaced with fresh complete growth medium after 24 h.

### IF

Paraffin-embedded tissues were cut into 4-mm-thick sections, deparaffinised and rehydrated in graded alcohol solutions. Antigen retrieval was then performed in citrate buffer (pH 6.0) for 15 min(s) The sections were incubated with an anti-TSC2 antibody or an anti-LC3 antibody overnight at 4°C. The next day, the sections were incubated with donkey anti-rabbit secondary antibodies. The nuclei were stained with Hoechst 34580 (63493; Sigma) at room temperature for 30 min. Fluorescence images were acquired at 1-μm intervals in the z-axis with an SP8 confocal microscope (Leica Microsystems, Wetzlar, Germany). The primary antibodies are listed in Supplementary Table 1.

### Western Blotting

Proteins were extracted from cells and tissues as previously described (Zhao et al., 2018). Next, the samples were subjected to SDS-PAGE and ultimately detected using an enhanced chemiluminescence (ECL) detection system (Pierce, Rockford, IL, United States). All antibodies used in the present study are listed in Supplementary Table 1.

### Cell Proliferation Assay

Cell proliferation ability was assessed using a cell density assay and an EdU staining assay. For the cell density assay, the cells were treated under different conditions for 24 and 48 h. Then, cell density was determined using a cell counter. For the EdU assay (40279ES25; Yeason, Shanghai, China), the cells were treated with EdU under different conditions for 2 h. Then, the cells were washed with PBS 3 times and cultured for an additional 48 h. After that, the cells were fixed with 4% paraformaldehyde and stained in accordance with the manufacturer’s instructions. The percentage of EdU-positive cells was calculated as the number of EdU-stained cells/total stained cells in the field.

### Cell Migration Assay

Twenty-four-well Transwell chambers (140640; Millipore, Billerica, MA, United States) were used for a cell migration assay in accordance with the manufacturer’s protocol. Briefly, 1 × 10^5^ cells in serum-free DMEM/F12 were added to the upper chamber of the insert, and 500 μL of DMEM/F12 containing 10% FBS was added to the lower chamber for culture. The cells were cultured in a humidified atmosphere for 24 h at 37°C under 5% CO_2_. The cells were then fixed with 4% paraformaldehyde for 15 min(s) and stained using 0.5% crystal violet (C8470; Solarbio, Beijing, China) for 20 min(s). The cells in the upper chamber were removed using a cotton swab, and the cells in the lower chamber in 3–4 random fields were counted under an inverted microscope (Leica Microsystems, Germany).

### Cell Scratch Wound Healing Assay

Cells were plated at a density of 2 × 10^5^ cells per well in a 6-well plate and cultured overnight at 37°C with 5% CO_2_. When the cells reached 80% confluence, they were treated as described, and after 72 h, a straight-line scratch was made on a confluent monolayer of cells using a sterile 1000-μL disposable pipette tip. The cells were washed with 1 mL of DMEM/F12 to remove debris and smooth the edge of the scratch. Images of cell migration were obtained using an inverted microscope (Leica Microsystems, Germany), and the gaps were calculated manually at 0 and 48 h post-scratch from nine random images for each group.

### Transmission Electron Microscopy

Transmission electron microscopy was performed as previously described (Gu et al., 2019). Briefly, Ishikawa cells were harvested and then fixed with 3% glutaraldehyde and 2% paraformaldehyde in 0.1 M PBS for 30 min at room temperature. The cells were post-fixed with 1% osmium tetroxide for 2 h and then stained with 3% aqueous uranyl acetate for 1 h at 37°C. The cells were dehydrated and then cut into ultrathin sections using an ultramicrotome. The samples were observed with a transmission electron microscope (Hitachi TEM system, Tokyo, Japan).

### Autophagy Assay

Autophagosome activity was labelled with a specific dye using an autophagy assay kit (MAK138; Sigma). Images were obtained at 1-μm intervals in the z-axis with an SP8 confocal microscope, and fluorescence intensity was measured using NIH/ImageJ software.

### Statistical Analysis

The data are presented as the mean ± SEM or as the median (IQR). Statistical results were obtained using GraphPad Prism 5. An unpaired two-tailed *t* test, a Mann-Whitney *U* test, Pearson’s χ^2^ test, Fisher’s exact test, two-way *ANOVA* or Pearson’s correlation coefficient analysis was applied to determine statistical significance. Not significant (NS), ^∗^, ^∗∗^, and ^∗∗∗^ indicate *p* > 0.05, *p* ≤ 0.05, *p* ≤ 0.01, and *p* ≤ 0.001, respectively.

## Discussion

The human endometrium is a dynamic tissue that undergoes monthly cyclic changes through a process dependent on the concentrations of sex hormones. Recent studies have reported that autophagy plays an important role in cyclic remodelling of the human endometrium (Markee, 1978). Although the expression of autophagy-associated proteins in human endometrial tissues has been described previously (Sales et al., 2004; Ren et al., 2010), to our knowledge, this is the first study to report the involvement of TSC2 expression in the cyclic remodelling of the human endometrium during the menstrual cycle. We examined TSC2 expression in human eutopic endometrial tissues according to the phases of the menstrual cycle. Previously, a similar pattern was reported: autophagy induction was increased in the epithelium in the secretory phase and menstruating endometrium, whereas autophagy induction was decreased in the proliferative phase (Choi et al., 2012). However, our results showed that in patients with AM, rhythmic expression of TSC2 was not observed, and epithelial TSC2 expression was significantly decreased in the secretory phase. Moreover, we confirmed the changes in autophagy induction in paired lesions and eutopic specimens by examining LC3A expression and found that the expression pattern of LC3A is positively correlated with TSC2 expression in the endometrium. These findings suggest that TSC2 is present primarily in the epithelial cells of the secretory endometrium and that it may be involved in endometrial cell autophagy during the menstrual cycle.

In line with the results obtained *in vivo*, the results of our *in vitro* experiments showed that downregulation of TSC2 in endometrial cells caused inhibition of autophagy through activation of the mTOR1 signalling pathway, leading to excessive proliferation, migration, and EMT. These effects were opposite those of TSC2 overexpression. Interestingly, EMT and migration, but not proliferation, could be enhanced by decreasing autophagy induction using rapamycin combined with SAR-405 *in vitro*. To support further research, a mouse model of AM should be constructed and used to detect the effects of TSC2 *in vivo*. Recently, a study has reported that mice with uterine-specific deletion of *Tsc2* exhibit myometrial proliferation by 12 and 24 weeks (Prizant et al., 2013). In ovariectomised Tsc2-null mice, myometrial growth is restored by E_2_ but not by P_4_, which indicates that disrupted synergy of steroid hormones may result in myometrial proliferation. In another study, a link among loss of TSC2, activation of mTORC1, and uterine leiomyomas was established with the Eker rat, which lacks one of the Tsc2 alleles. Almost 72% of aged female Eker rats develop microscopic uterine leiomyoma-like lesions (Eker et al., 1981). Although mice with conditional TSC2 knockout do not exhibit typical glandular epithelial invasion of the myometrium, these selectively bred and genetically modified animal models exhibit obvious features of AM, such as myometrial hyperplasia (Kaneko-Tarui et al., 2014).

Finally, we demonstrated that compared with cells treated with E_2_ alone, both Ishikawa cells and nHEECs treated with E_2_ combined with P_4_ (to mimic the secretory phase) exhibited increased TSC2 expression (Supplementary Figures 1A,B), inactivation of mTOR1 signalling and increased autophagy induction. These findings are consistent with previous observations showing that autophagy induction increases in normal endometrial cells following the combined release of ovarian steroids (Choi et al., 2012, 2014; Ruiz et al., 2016). These results also suggest that autophagy in normal endometrial cells can be induced by mTOR1 inhibition in the secretory and menstrual phases. mTOR1 is a serine/threonine protein kinase that regulates cellular processes such as cell proliferation, growth, and autophagy (Long et al., 2004). In endometriosis, inadequate responses to P_4_ in eutopic and ectopic endometrial cells and tissues contribute to increased cell proliferation, in which mTOR1 is involved (Al-Sabbagh et al., 2012). The ectopic endometrium in patients with endometriosis exhibits greater phosphorylation of mTOR1 than the eutopic endometrium (McKinnon et al., 2016). The present study further demonstrated that TSC2 expression did not change in aHEECs following treatment with ovarian steroids. Thus, autophagy may not be induced in aHEECs due to disinhibition of mTOR1 activity during the secretory and menstrual phases. This possibility was supported by the finding that a higher level of mTOR1 activity, which inhibited autophagy induction, was present in aHEECs following withdrawal of E_2_ and P_4_ than in nHEECs. Taken together, our results verify that downregulation of TSC2 in epithelial cells results in inhibition of autophagy through activation of the mTOR1 signalling pathway.

In conclusion, our results demonstrate a crucial role of TSC2 in migration and EMT in endometrial cells. Hypo-expression of TSC2 enhances EMT and migration induced by ovarian steroids in endometrial cells *via* the mTOR1-autophagy axis. These findings provide great insight to enhance our understanding of the pathophysiology of AM and to aid in the development of a new therapeutic target for AM.

## Data Availability Statement

The original contributions presented in the study are included in the article/Supplementary Material, further inquiries can be directed to the corresponding author.

## Ethics Statement

The studies involving human participants were reviewed and approved by the Ethics Committee of the International Peace Maternity and Child Health Hospital (approval No. GKLW2017-71). The patients/participants provided their written informed consent to participate in this study.

## Author Contributions

HX conceived and designed the study. N-HG performed the experiments, analysed the data, and wrote the manuscript. G-JL participated in the immunostaining and cell culture. B-XY participated in the analysis of demographics. MY, YL, and FS provided reagents and comments for the experiments. All authors gave final approval for publication and agreed to be held accountable for the work described herein.

## Conflict of Interest

The authors declare that the research was conducted in the absence of any commercial or financial relationships that could be construed as a potential conflict of interest.

## Publisher’s Note

All claims expressed in this article are solely those of the authors and do not necessarily represent those of their affiliated organizations, or those of the publisher, the editors and the reviewers. Any product that may be evaluated in this article, or claim that may be made by its manufacturer, is not guaranteed or endorsed by the publisher.

## Supplementary Material

Supplementary Figure 1 P_4_ combined with E_2_ can increase the expression of TSC2 in the Ishikawa cell line. **(A,B)** Representative immunoblots and quantification of TSC2 in Ishikawa cells treated with hormones. The data are presented as the mean ± SEM. NS *p* > 0.05, ^∗^*p* < 0.05, ^∗∗^*p* < 0.01, and ^∗∗∗^*p* < 0.001 compared with the E_2_ group.

Supplementary Figure 2 Autophagy inhibition does not affect TSC2 expression or mTOR activation. **(A,B)** Representative immunoblots and quantification of LC3, phosphorylated mTOR1 and TSC2 in Ishikawa cells under various conditions. The data are presented as the mean ± SEM. NS *p* > 0.05, ^∗^*p* < 0.05, ^∗∗^*p* < 0.01, and ^∗∗∗^*p* < 0.001 compared with the mock + E_2_ group.

Supplementary Figure 3 TSC2 overexpression promotes autophagy induction and inhibits proliferation in endometrial cells. **(A)** Typical images of autophagic vacuoles detected using immunofluorescence staining and transmission electron microscopy (TEM) (the arrows indicate autophagosomes) and EdU-positive cells. Immunofluorescence scale bars, 25 μm; TEM scale bars, 1 μm; EdU scale bars, 50 μm. **(B)** Fluorescence intensity of the autophagic vacuoles in panel **(A)**. **(C)** Statistical results showing the percentage of EdU-positive cells in the EdU incorporation assay. **(D)** Cell growth curves of Ishikawa cells under various conditions. The data are presented as the mean ± SEM. NS *p* > 0.05, ^∗^*p* < 0.05, ^∗∗^*p* < 0.01, and ^∗∗∗^*p* < 0.001 compared with the mock + E_2_ group.

Supplementary Figure 4 TSC2 overexpression promotes the migration and EMT of endometrial cells. **(A–C)** Representative images **(A,B)** and statistics **(C)** of cells in the scratch assay and Transwell assay. Scale bars in **(A,B)**, 100 μm **(B)**. **(D,E)** Representative immunoblot and quantification of the expression of N-Cadherin, E-Cadherin, Snail and Slug under various conditions. The data are presented as the mean ± SEM. NS *p* > 0.05, ^∗^*p* < 0.05, ^∗∗^*p* < 0.01, and ^∗∗∗^*p* < 0.001 compared with the mock + E_2_ group.

Supplementary Figure 5 mTOR inhibition promotes autophagy but does not induce hyper-expression of TSC2. **(A,B)** Representative immunoblots and quantification of LC3, phosphorylated mTOR1 and TSC2 in Ishikawa cells under various conditions. The data are presented as the mean ± SEM. NS *p* > 0.05, ^∗^*p* < 0.05, ^∗∗^*p* < 0.01, and ^∗∗∗^*p* < 0.001 compared with the sh + E_2_ group.
