# Supplementary figures and images for: Hypo-Expression of Tuberin Promotes Adenomyosis via the mTOR1-Autophagy Axis
Source: Front Cell Dev Biol. 2021 Jul 29;9:710407. doi: 10.3389/fcell.2021.710407 (PMC8358309; doi:10.3389/fcell.2021.710407)

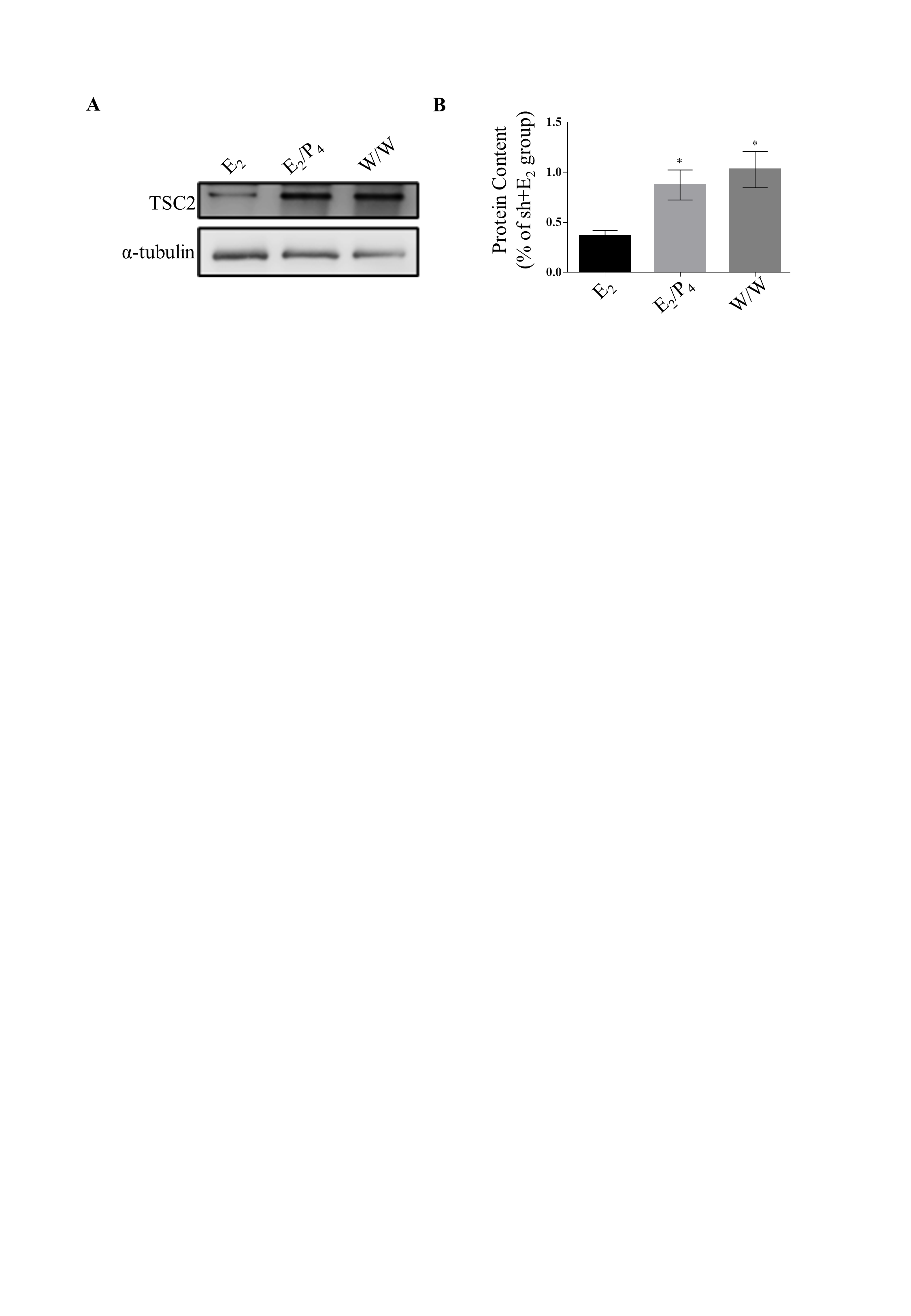

Supplement: Supplementary Figure 1 — P4 combined with E2 can increase the expression of TSC2 in the Ishikawa cell line. (A,B) Representative immunoblots and quantification of TSC2 in Ishikawa cells treated with hormones. The data are presented as the mean ± SEM. NS p > 0.05, ∗p < 0.05, ∗∗p < 0.01, and ∗∗∗p < 0.001 compared with the E2 group. [file Image_1.TIF]

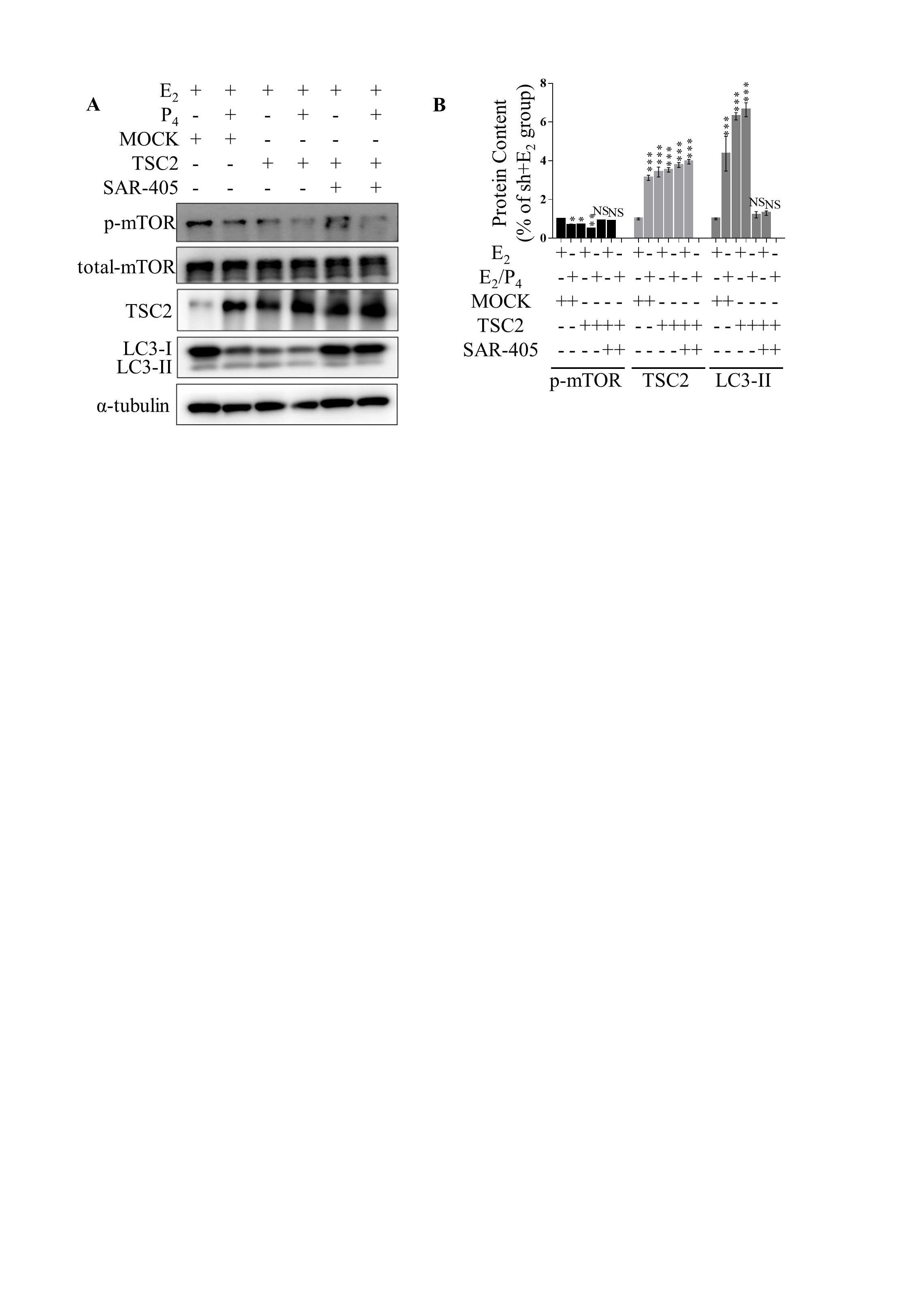

Supplement: Supplementary Figure 2 — Autophagy inhibition does not affect TSC2 expression or mTOR activation. (A,B) Representative immunoblots and quantification of LC3, phosphorylated mTOR1 and TSC2 in Ishikawa cells under various conditions. The data are presented as the mean ± SEM. NS p > 0.05, ∗p < 0.05, ∗∗p < 0.01, and ∗∗∗p < 0.001 compared with the mock + E2 group. [file Image_2.TIF]

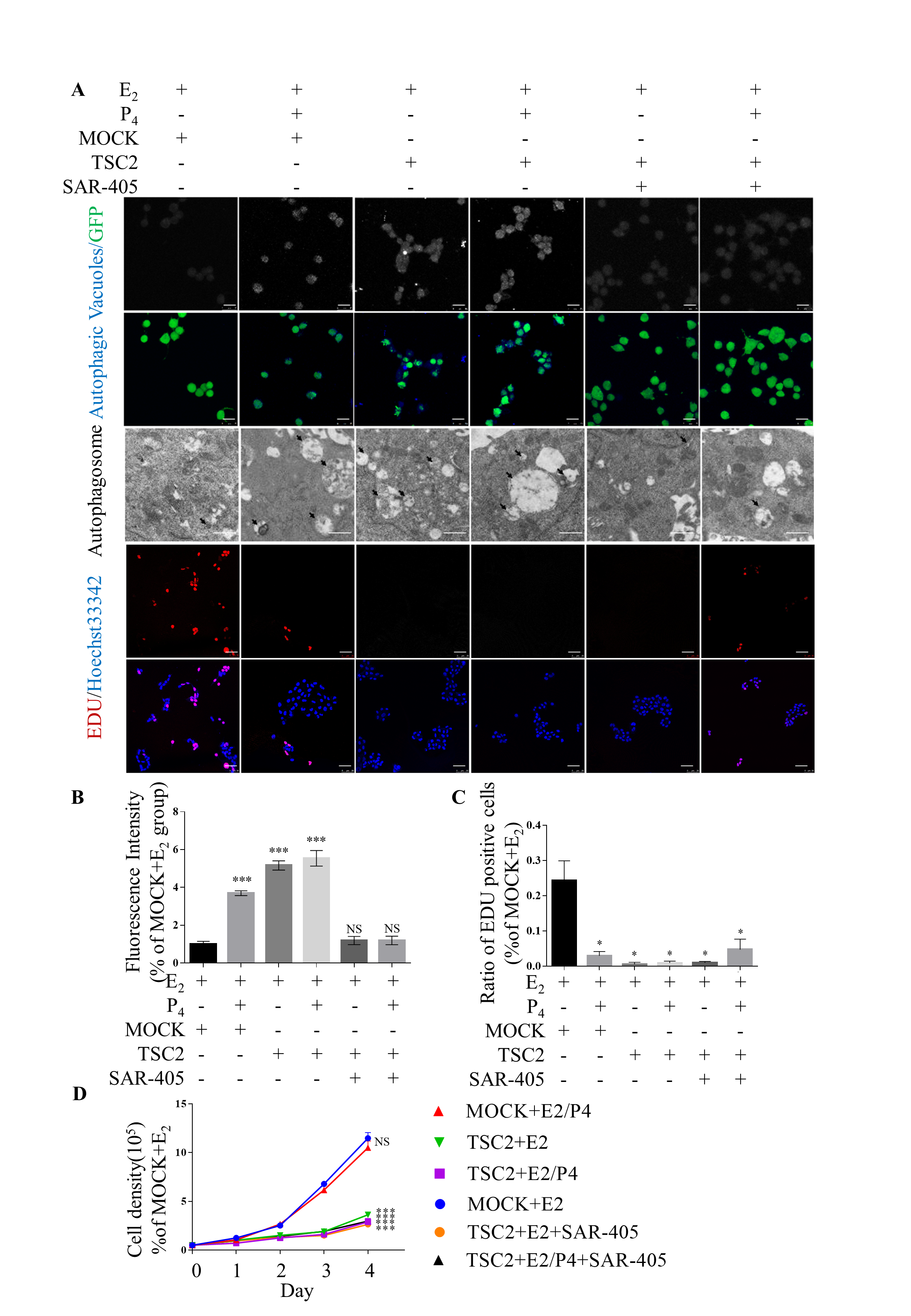

Supplement: Supplementary Figure 3 — TSC2 overexpression promotes autophagy induction and inhibits proliferation in endometrial cells. (A) Typical images of autophagic vacuoles detected using immunofluorescence staining and transmission electron microscopy (TEM) (the arrows indicate autophagosomes) and EdU-positive cells. Immunofluorescence scale bars, 25 μm; TEM scale bars, 1 μm; EdU scale bars, 50 μm. (B) Fluorescence intensity of the autophagic vacuoles in panel (A). (C) Statistical results showing the percentage of EdU-positive cells in the EdU incorporation assay. (D) Cell growth curves of Ishikawa cells under various conditions. The data are presented as the mean ± SEM. NS p > 0.05, ∗p < 0.05, ∗∗p < 0.01, and ∗∗∗p < 0.001 compared with the mock + E2 group. [file Image_3.TIF]

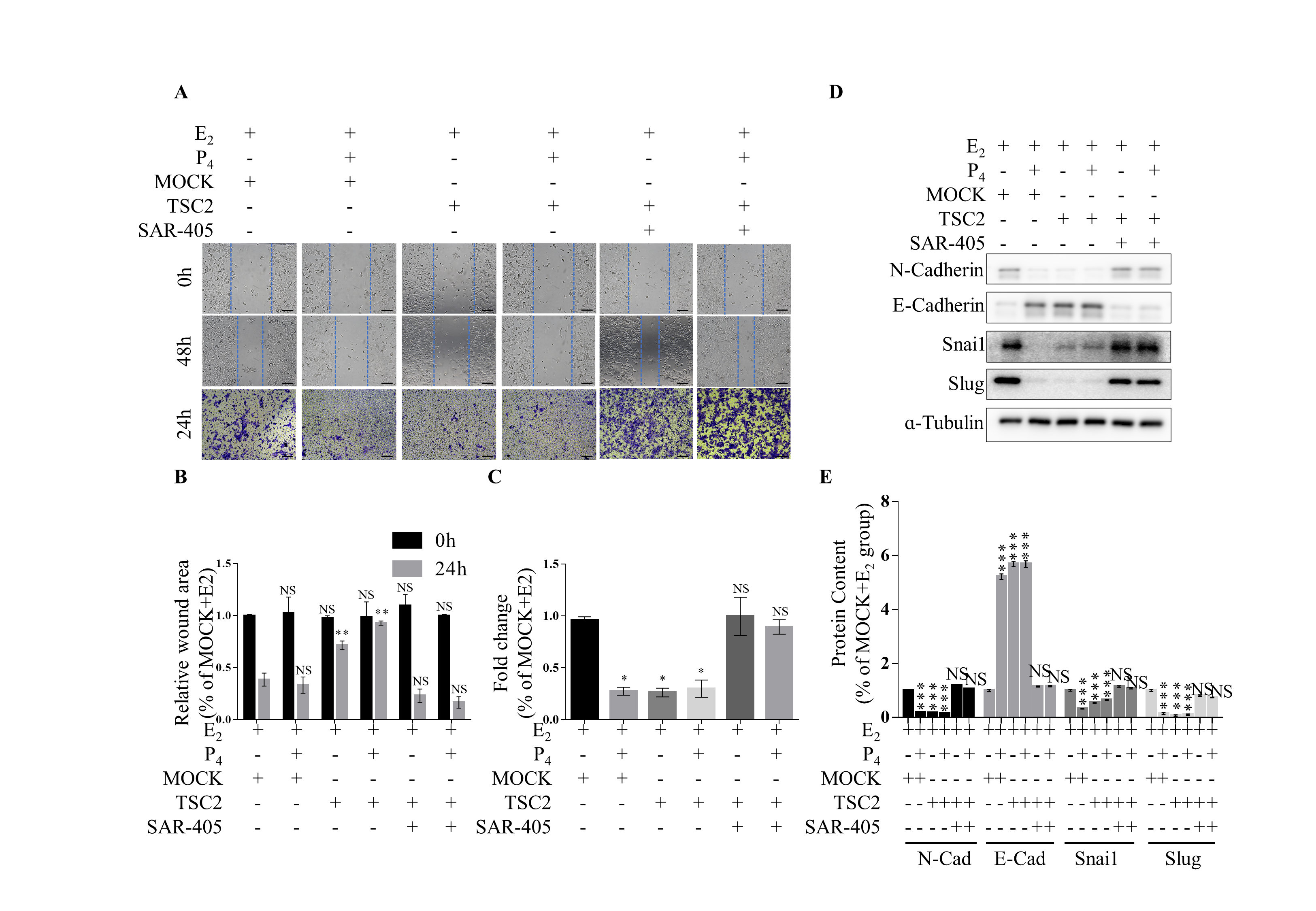

Supplement: Supplementary Figure 4 — TSC2 overexpression promotes the migration and EMT of endometrial cells. (A–C) Representative images (A,B) and statistics (C) of cells in the scratch assay and Transwell assay. Scale bars in (A,B), 100 μm (B). (D,E) Representative immunoblot and quantification of the expression of N-Cadherin, E-Cadherin, Snail and Slug under various conditions. The data are presented as the mean ± SEM. NS p > 0.05, ∗p < 0.05, ∗∗p < 0.01, and ∗∗∗p < 0.001 compared with the mock + E2 group. [file Image_4.TIF]

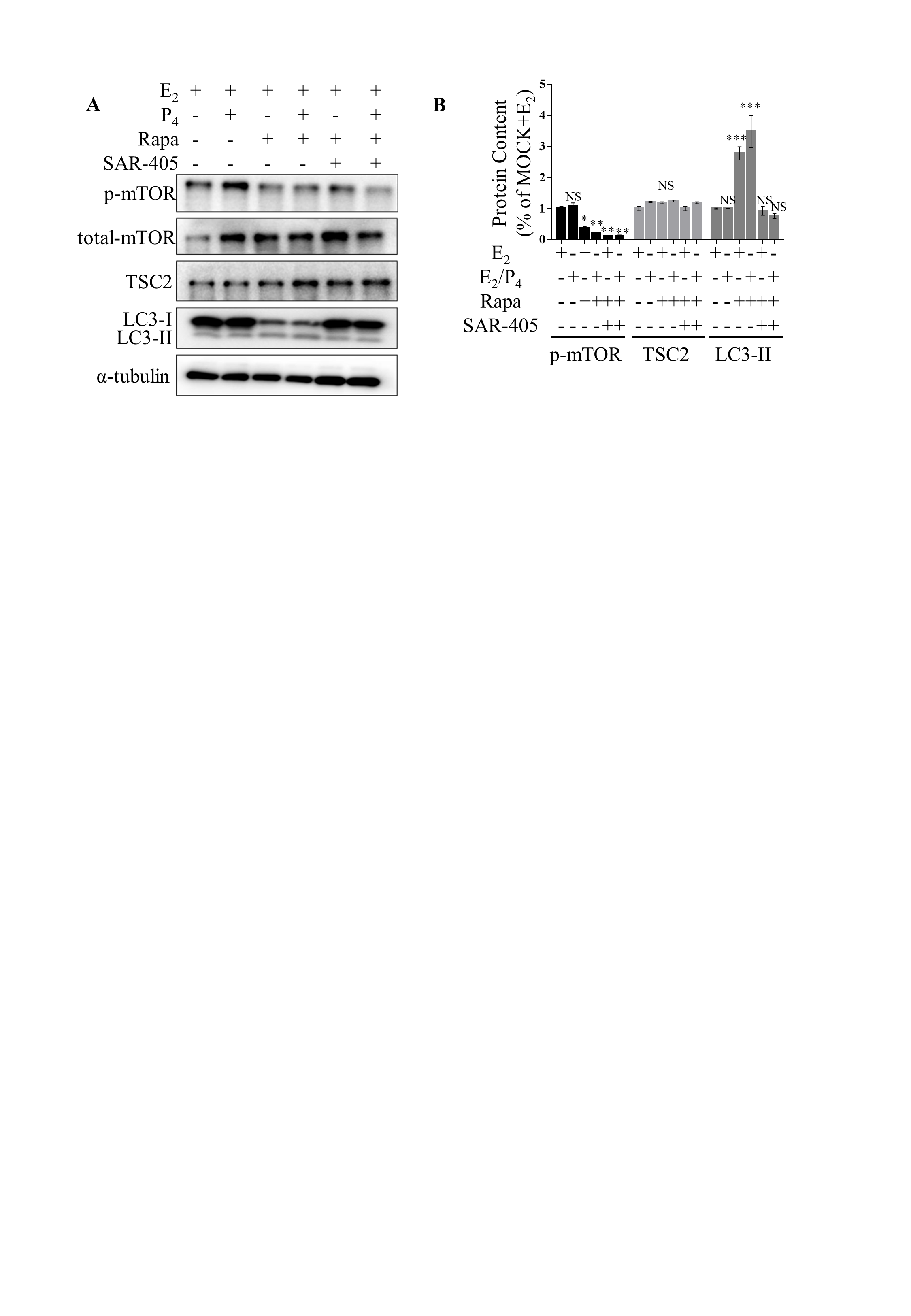

Supplement: Supplementary Figure 5 — mTOR inhibition promotes autophagy but does not induce hyper-expression of TSC2. (A,B) Representative immunoblots and quantification of LC3, phosphorylated mTOR1 and TSC2 in Ishikawa cells under various conditions. The data are presented as the mean ± SEM. NS p > 0.05, ∗p < 0.05, ∗∗p < 0.01, and ∗∗∗p < 0.001 compared with the sh + E2 group. [file Image_5.TIF]
